# Preloading magnesium attenuates cisplatin-associated nephrotoxicity: pilot randomized controlled trial (PRAGMATIC study)

**DOI:** 10.1016/j.esmoop.2021.100351

**Published:** 2021-12-23

**Authors:** S. Suppadungsuk, W. Phitakwatchara, T. Reungwetwattana, A. Pathumarak, B. Phakdeekitcharoen, C. Kitiyakara, P. Srisuwarn, A. Davenport, A. Nongnuch

**Affiliations:** 1 Chakri Naruebodindra Medical Institute, Faculty of Medicine Ramathibodi Hospital, Mahidol University, Samut Prakan, Thailand; 2 The 50^th^ Anniversary Mahavajiralongkorn Hospital, Ubon Ratchathani, Thailand; 3 Division of Medical Oncology, Department of Medicine, Faculty of Medicine Ramathibodi Hospital, Mahidol University, Bangkok, Thailand; 4 Division of Nephrology, Department of Medicine, Faculty of Medicine Ramathibodi Hospital, Mahidol University, Bangkok, Thailand; 5 Division of Internal Medicine, Department of Medicine, Faculty of Medicine Ramathibodi Hospital, Mahidol University, Bangkok, Thailand; 6 UCL Centre for Nephrology, Royal Free Hospital, University College London, London, UK

**Keywords:** cisplatin nephrotoxicity, acute kidney disease, magnesium preloading

## Abstract

**Background:**

Cisplatin is one of the most potent chemotherapeutic drugs used in head and neck cancer treatment; however, nephrotoxicity is the major side-effect limiting usage. Magnesium supplementation has been reported to reduce risk in non-controlled studies. We investigated whether preloading with magnesium prevents nephrotoxicity with a low-dose weekly cisplatin regimen.

**Methods:**

We carried out a prospective pilot, single-blinded, randomized controlled trial to compare cisplatin-associated acute kidney injury (cis-AKI) and acute kidney disease (cis-AKD) between two groups: intravenous 0.9% NaCl 500 ml + KCL 20 mEq over 4 h pre-cisplatin 40 mg/m^2^ weekly for 7-8 weeks (control group) compared with additional 16 mEq magnesium added to the saline infusion (Mg group) in 30 head and neck cancer patients. Cis-AKI was defined as an increased serum creatinine (SCr) ≥ 0.3 mg/dl within 7 days and cis-AKD is an increased SCr ≥ 0.3 mg/dl between last SCr and baseline pre-chemotherapy SCr.

**Results:**

The overall cisplatin tumor response rate and survival were comparable between groups. The baseline characteristics were comparable between groups, although SCr was lower in the controls (0.70 ± 0.17 versus 0.87 ± 0.17 mg/dl, *P* = 0.01). The incidence of cis-AKI was similar (4.6% versus 1.3%); however, the incidence of cis-AKD was higher for the control group (46.7% versus 6.7%, hazard ratio = 0.082, 95% confidence interval 0.008-0.79, *P* = 0.03). The time to develop cis-AKD was significantly shorter in the control group (*P* = 0.007).

**Conclusions:**

The magnesium-preloading regimen was safe and significantly showed a decreased incidence of cis-AKD. The encouraging results of our pilot study need to be confirmed in a large-scale randomized controlled trial.

## Highlights

• The magnesium preloading regimen tend to lower incidence of cisplatin-associated acute kidney injury (cis-AKI).
• The effects of cisplatin on kidney function were mainly subacute, thus clinicians should carefully monitor not only for cis-AKI but also cis-AKD.

## Introduction

Cisplatin-based treatments remain the standard of care for patients with head and neck cancers; however, the acute, subacute and longer-term toxicities of cisplatin limit usage. Nephrotoxicity is one of the most common complications, accounting for 20%-50%, depending upon definitions of nephrotoxicity and cumulative cisplatin dosage.^1^, ^2^, ^3^, ^4^ Cisplatin nephrotoxicity may present as not only acute and subacute kidney injury or chronic kidney disease, but also with electrolyte abnormalities, characterized by hypokalemia and hypomagnesemia.^5^ The pathogenesis of nephrotoxicity may result from a direct toxic effect of cisplatin accumulation in the proximal tubular epithelial cells (PTEC), as well as secondary to the inflammatory effects of cisplatin, with increased generation of reactive oxygen species and inflammatory mediators leading to PTEC necrosis and apoptosis, leading to hypomagnesemia, hypokalemia and a reduction in kidney function.^5^

Experimental animal models have demonstrated that magnesium depletion increases renal tubular platinum accumulation, oxidative stress and tubular injury leading to a reduction in kidney function. Moreover, in a mouse model of magnesium depletion, magnesium supplementation was shown to reduce renal tubular injury following both a single dose and also multiple doses of cisplatin.^6^, ^7^, ^8^ Most importantly, magnesium supplementation did not reduce the therapeutic effect of cisplatin on tumor cells.^7^^,^^9^

A retrospective analysis of cancer registry data has suggested that magnesium supplementation has a protective effect on kidney function compared to pre-treatment with 0.9% NaCl alone.^1^^,^^2^^,^^4^^,^^10^ Studies have varied in the amount of magnesium given, the timing of magnesium administration in relation to cisplatin and forced diuresis protocols. For example, one study gave magnesium in an oral replacement fluid after cisplatin administration^11^ and another regular oral magnesium supplementation.^12^ Although these studies reported that magnesium supplementation reduced kidney injury in the short term, the definition of kidney injury varied. To overcome the variability in definitions of kidney injury, three categories have been proposed according to the time between baseline serum creatinine (SCr) and subsequent increase in SCr ≥ 0.3 mg/dl, defining acute kidney injury (AKI) with the increase in SCr within 7 days, acute kidney disease (AKD) with the increase in SCr between 7 and 90 days and chronic kidney disease (CKD) with the increase in SCr persisting for >90 days.^13^

Due to the potential toxicity of cisplatin, different dosing schedules have been investigated in locally advanced head and neck cancer—both nasopharyngeal cancer (NPC) and non-NPC. Chemoradiation with cisplatin 100 mg/m^2^ given once every 3 weeks is the standard of care in the past decades. However, low-dose once-a-week cisplatin (30-40 mg/m^2^) is increasingly substituted because of perceived lower toxicity, convenience and omitting the hospital in-patient stay. There were several randomized phase II and phase III studies in India, Japan and Korea that demonstrated the non-inferiority efficacy of low-dose once-a-week cisplatin compared to 3-weekly cisplatin in either post-operative concurrent chemoradiation therapy (CCRT) or definitive CCRT in both NPC and non-NPC.^14^, ^15^, ^16^ However, studies reported from Mumbai showed the benefit of locoregional control rate in patients treated with once-every-3-weeks cisplatin, but the progression-free survival and overall survival (OS) were similar between these different dosing schedules.^15^

A review of studies reporting on nephrotoxicity with standard high-dose cisplatin has led to clinical practice guidelines recommending that 8-20 mEq of magnesium should be administered intravenously prior to standard high-dose cisplatin administration.^1^^,^^3^^,^^17^, ^18^, ^19^ Minzi et al.^17^ reported that 8 mEq of intravenous magnesium decreased the incidence of nephrotoxicity without serious side-effects. In addition, Hase and colleagues^19^ demonstrated the safety and efficacy of an additional 20 mEq magnesium compared to 0.9% NaCl and mannitol for standard high-dose cisplatin regimens (≥60 mg/m^2^ every 3-4 weeks). Moreover, Willox et al.^20^ reported that 16 mEq of magnesium administered either orally or intravenously reduce renal tubular damage markers with low dose of cisplatin (20 mg/m^2^), and Yamamoto and colleagues^2^ reported significantly decreased kidney function with 0.9% NaCl hydration compared to 15 mEq magnesium with low-dose cisplatin (40 mg/m^2^) weekly for 6 weeks. Based on our review of a published series, we decided to study the effect of an additional 16 mEq magnesium to 0.9% NaCl 500 ml due to reported safety profiles and availability of 10% MgSO_4_ preparation in our hospital.

As such we undertook a pilot study to determine the effect of preloading with additional magnesium 16 mEq compared to standard care with 0.9% NaCl on the incidence of AKI and AKD in head and neck cancer patients receiving 7-8 weeks of a low-dose cisplatin regimen (40 mg/m^2^).

## Materials and methods

### Study population

This study is a prospective randomized (1 : 1), single-blinded (patients blinded), parallel controlled, single-center study. Head and neck cancer patients who received concurrent chemotherapy with cisplatin 40 mg/m^2^ weekly for 7-8 weeks at Ramathibodi hospital, Bangkok, Thailand, from April 2015 to March 2016 were enrolled in this clinical trial. The inclusion criteria were age ≥18 years old, Eastern Cooperative Oncology Group performance status 0-2, serum creatinine ≤1.5 mg/dl or estimated glomerular filtration rate (eGFR) using the chronic kidney disease epidemiology collaboration (CKD EPI) formula ≥60 ml/min per 1.73 m^2^. The exclusion criteria were previous cisplatin treatment, uncontrolled intercurrent illness and pregnancy.

Our sample size calculation for this pilot study was based on the effect of magnesium preloading reducing AKI (primary outcome) by 20% compared to the 0.9% NaCl group. This gave a total number of patients of 100, with power to detect the hypothesized difference between the two groups (two-sided α = 0.05).

### Study protocol and data collection

After study enrollment, patients were assigned to their group—control saline group and magnesium-supplemented (Mg group) group—using sealed opaque envelopes that had a randomization number of block sizes of two. The study was single blinded, with patients blinded, whereas the investigating physicians and oncologists were not. Patients were randomly assigned in a 1 : 1 ratio to either the 0.9% NaCl group (control group) who had 0.9% NaCl 500 ml + KCL 20 mEq administered intravenously over 4 h (125 ml/h) before every cisplatin dose or the Mg group who were given an additional 10% MgSO_4_ 16 mEq (8 mmol) with the 0.9% NaCl 500 ml + KCL 20 mEq. At every visit, blood was collected for blood count, serum sodium, potassium, magnesium, creatinine, blood urea nitrogen and, if there were abnormal values, then toxicity was graded and managed according to the Common Terminology Criteria for Adverse Events (CTCAE version 5). The oncologists switched cisplatin to carboplatin if patients could not tolerate cisplatin toxicities—nausea and vomiting grade 2-3 which did not resolve despite treatment with appropriate anti-nausea and vomiting medications, persistent increase in serum creatinine >1.5× upper normal limit, and persistent electrolyte imbalance (grade 2-3) despite treatments designed to correct electrolytes prescribed by nephrologists.

### Outcomes

Our primary outcome was the incidence of cisplatin-associated AKI (cis-AKI), defined as an acute increase in serum creatinine (SCr) ≥ 0.3 compared to baseline SCr from the previous week, with a secondary outcome of the incidence of cisplatin-associated AKD (cis-AKD), defined as an increase in serum creatinine ≥ 0.3 between the pre-cisplatin baseline SCr and last SCr. The last SCr was defined as the SCr 1-4 weeks after completion of the treatment course of cisplatin or last SCr when patients dropped out from cisplatin treatment.

### Ethics

All patients provided written informed consent and the study protocol was approved by the Ethics Committee, Ramathibodi Hospital (MURA 2015/172) and conducted according to the principles of the declaration of Helsinki. This study was registered with ClinicalTrials.gov with a trial identification number of NCT02481518.

### Statistical analysis

We collected and analyzed all endpoint data in accordance with the intention-to-treat principle. For baseline characteristics, continuous data are presented as mean ± standard deviation for normally distributed data and median (interquartile range) for non-parametric data. The independent sample *t*-test and Mann–Whitney *U* test were used to determine the difference between groups. Categorical data are presented as number (%), and Pearson’s chi-square test was used to compare categorical data. The linear mixed model was used to determine the difference between baseline and subsequent values within the same groups. The cumulative incident of AKD was plotted using a Kaplan–Meier curve. Overall survival calculated with Kaplan–Meier survival curves, used the date of diagnosis and the date of death from any cause. Data were analyzed and plotted using statistical analysis in social science (SPSS version 25, IBM, Armonk, NY). A *P* value <0.05 was considered statistically significant.

## Results

### Enrollment

**Figure 1:**
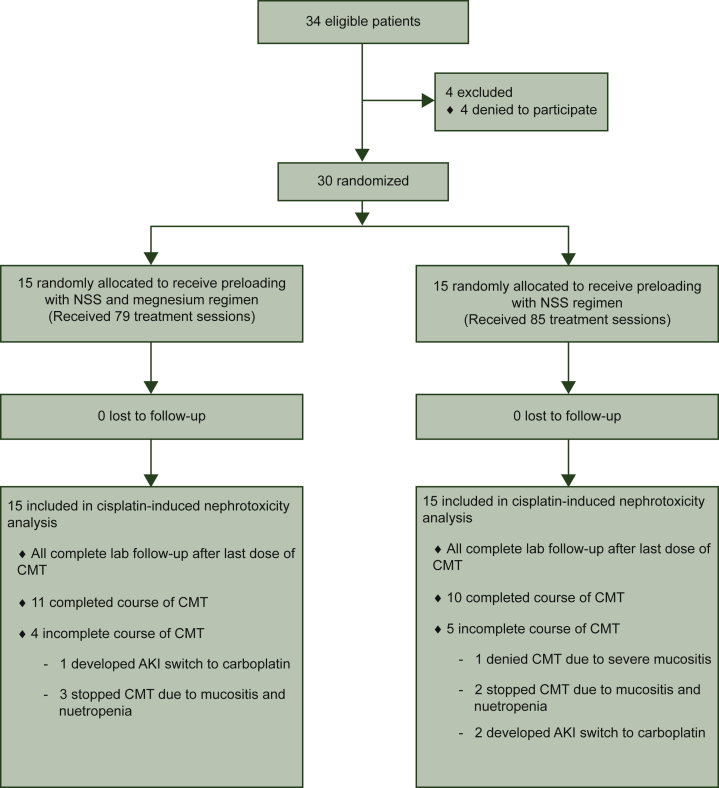
Study Consolidated Standards of Reporting Trials (CONSORT) diagram. Number of patients who were recruited to the study, assigned to a study group and completed the protocol. AKI, acute kidney injury; CMT, chemotherapy; NSS, 0.9% NaCl.

Between April 2015 and March 2016, 34 patients were eligible for the study and 4 patients declined to participate; thus 30 patients were enrolled. The Consolidated Standards of Reporting Trials (CONSORT) flow diagram is presented in Figure 1.

### Baseline characteristics

The majority of baseline characteristics of the patients in the two groups were comparable (Table 1). The Mg group were marginally older, and there were fewer female patients, but these differences were not significant. Baseline pre-cisplatin SCr was higher in the Mg group, while the eGFR was not different. Although the Mg group had a lower baseline serum bicarbonate and higher serum chloride, and lower diastolic blood pressure, these differences became non-significant after adjustment for multiple statistical testing.

**Table 1:** Baseline characteristics at study inclusion

Parameters	0.9% NaCl group (*N* = 15)	Magnesium group (*N* = 15)	*P* value
Age (years)	49.1 ± 9.3	55.3 ± 13.5	0.15
Female sex, *n* (%)	5 (33.3)	2 (13.3)	0.2
Hypertension, *n* (%)	2 (13.3)	2 (13.3)	1.0
Diabetes, *n* (%)	3 (20)	3 (20)	1.0
Body mass index (kg/m^2^)	24.1 ± 3.5	23.0 ± 4.1	0.44
Systolic blood pressure (mmHg)	118 ± 10	115 ± 10	0.44
Diastolic blood pressure (mmHg)	74 ± 6	68 ± 9	0.04
Administered ACEi^a^, *n* (%)	1 (6.6)	1 (6.6)	1.0
Hemoglobin (g/dl)	12.9 ± 1.2	12.5 ± 1.7	0.45
Hematocrit (%)	39.8 ± 4.5	37.8 ± 4.8	0.26
Total white cell count (cell/ul)	8309 ± 3518	8152 ± 2245	0.98
Serum sodium (mEq/l)	137.0 ± 3.95	137.5 ± 3.1	0.73
Serum potassium (mEq/l)	4.27 ± 0.48	4.16 ± 0.52	0.55
Serum chloride (mEq/l)	100.2 ± 4.8	103.3 ± 3.2	0.03
Serum bicarbonate (mEq/l)	26.7 ± 3.3	24.2 ± 2.3	0.04
Serum albumin (g/dl)	3.62 ± 0.57	3.65 ± 0.43	0.89
Serum magnesium (mg/dl)	2.23 ± 0.14	2.18 ± 0.29	0.61
Serum creatinine at baseline (mg/dl)	0.70 ± 0.17	0.87 ± 0.17	0.01
eGFR^b^ at baseline (ml/min)	103.6 ± 14.8	94.5 ± 15.8	0.12
Nasopharyngeal cancer (%)	5 (33.3)	3 (20)	0.41
Cycle of cisplatin median (IQR)	6 (3)	6 (2)	0.39
Cumulative cisplatin dosage (mg)	342.1 ± 126.3	334.7 ± 120.3	0.87

eGFR, estimated glomerular filtration rate; IQR, interquartile range.

a ACEi = angiotensin-converting enzyme inhibitors.

b eGFR was calculated by using chronic kidney disease epidemiology (CKD EPI) formula.

### Study treatment

During the study, nine (30%) patients were not able to complete the full course of cisplatin treatments. The cisplatin treatment course was not complete by five patients in the control group due to grade 3 mucositis (*n* = 1), grade 3 neutropenia (*n* = 2) and AKI (*n* = 2), and by four patients in the magnesium group due to grade 3 neutropenia (*n* = 3) and AKI (*n* = 1) (Figure 1). The median number of cisplatin cycles completed was six, and was similar for both groups and so the cumulative cisplatin dosages were comparable (Table 1).

**Figure 2:**
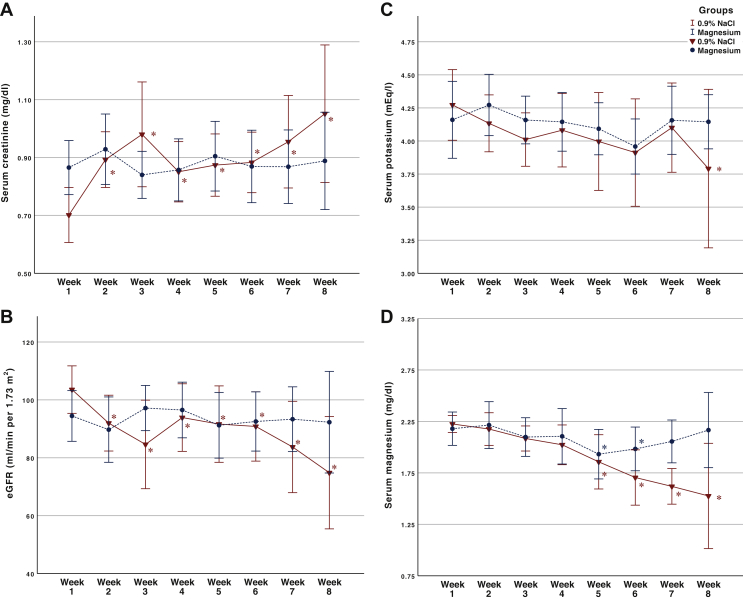
Mean serum creatinine (A), estimated glomerular filtration rate (eGFR) (B), serum potassium (C) and serum magnesium (D) during study period amongst treatment groups. ∗*P* < 0.05 compared to baseline value in linear mixed effect model.

The weekly SCr, eGFR, serum potassium and serum magnesium measurements are shown in Figure 2. Compared to the Mg group, there appeared to be a trend between weeks 5 and 6 for a fall in eGFR and serum magnesium, and an increase in serum creatinine in the control group, whereas there were no differences for serum potassium.

### Study outcomes

There were five episodes (3%) of cis-AKI in 164 treatment cycles, although higher in the control group (4.6% versus 1.3%), and this was not statistically significant. The hazard ratio (HR) for cis-AKI in the Mg group was 0.19 [95% confidence interval (CI) = 0.02-2.02, *P* = 0.2] (Table 2). The incident of cis-AKD was 26.7% which was significantly higher in the control group (46.7% versus 6.7%) and the HR for cis-AKD in the Mg group was 0.082 (95% CI = 0.008-0.79, *P* = 0.03).

**Table 2:** Primary and secondary outcomes of the study: incidence of acute kidney injury (AKI) and acute kidney disease (AKD)

	Total events (164 sessions, events in 30 patients), (%)	Events in the control group (85 sessions, events in 15 patients), (%)	Events in the magnesium group (79 sessions, events in 15 patients), (%)	Hazard ratio (95% confidence limit)	*P* value
Primary outcome					
Incidence of AKI (% of treatment session)	5 (3.0)	4 (4.6)	1 (1.3)	0.19 (0.02-2.02)	0.2
Secondary outcome					
Incidence of AKD (% of patients)	8 (26.7)	7 (46.7)	1 (6.7)	0.082 (0.008-0.79)	0.03

**Figure 3:**
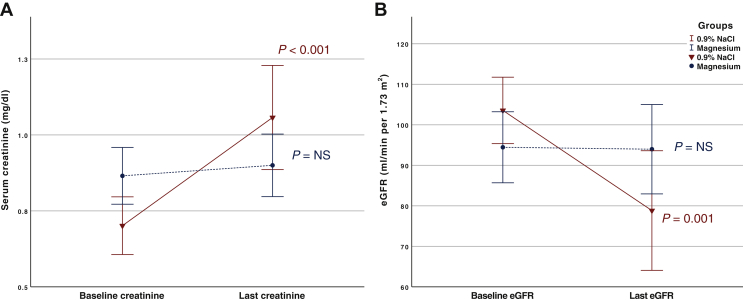
Mean serum creatinine changed compared to baseline creatinine and last creatinine (A) and mean estimated glomerular filtration rate (eGFR) change compared to baseline eGFR and last eGFR (B) amongst treatment groups. *P* value = paired *t*-test compared to baseline values.

When considering the difference in SCr between the baseline and last measurements (Figure 3), there was a significant increase in the control group (mean SCr increase 0.36, 95% CI = 0.19-0.52, *P* < 0.001), whereas SCr remained unchanged in the Mg group (Supplementary Figures S2-S4, available at https://doi.org/10.1016/j.esmoop.2021.100351). This was mirrored by a significant fall in eGFR in the control group (mean change in eGFR −24.7 ml/min per 1.73 m^2^, 95% CI = −36.8 to −12.6, *P* = 0.001) in contrast to no change in the Mg group. The time to develop cis-AKD was significantly shorter in the control group (*P* = 0.007), (Supplementary Figure S1, available at https://doi.org/10.1016/j.esmoop.2021.100351).

Importantly, the efficacy of cisplatin treatment was similar, both in terms of complete response rate (control 60 versus Mg group 64.7%) and median OS (mOS) for the two groups after CCRT. The mOS was longer in the Mg group compared to the controls. [57.6 versus 47.6 months, HR = 0.98, 95% CI = 0.36-2.71, *P* = 0.97], but not significantly so.

### Adverse events

The incidence of mucositis and neutropenia were non-significantly lower in the control group compared to the Mg group (14.1% versus 26.6%, *P* = 0.12 and 2.3% versus 5.1%, *P* = 0.31), respectively.

## Discussion

This pilot study is the first randomized controlled trial, with parallel comparison of supplementation with magnesium compared to a standard preloading regimen in low-dose weekly cisplatin chemotherapy for patients with head and neck cancer. Although the incidence of cis-AKI was not statistically different, the Mg-supplemented group had a significantly lower incidence of cis-AKD. Our original primary outcome had been the incidence of cis-AKI, and we had estimated the study sample size based on earlier data that relative risk of magnesium supplementation for preventing nephrotoxicity was ∼0.2, thus giving a study population of 100 patients. However, after the first 12 months the preliminary analysis of the first 30 patients showed that although the incidence of cis-AKI (primary outcome) was not different, there was a marked difference in the incidence of cis-AKD, our secondary outcome. These results were reviewed by the investigators and the clinical oncology team, and a decision was made to terminate the study due to the higher risk of cis-AKD in the control group.

Platinum-based chemotherapy is widely accepted as the first-line treatment for a number of different types of cancer. However, the serious adverse side-effects of cisplatin and their prevention have not been well studied. Cisplatin is reported to cause a wide range of kidney dysfunction ranging from AKI to subacute toxicity including AKD, CKD, renal tubular acidosis, renal tubular sodium wasting and concentrating defects and electrolyte abnormalities, typified by hypomagnesemia, and hypokalemia. On the one hand, cisplatin can induce proximal tubular dysfunction with a Fanconi-like syndrome, but on the other hand hypomagnesemia potentiates cisplatin accumulation in tubular cells leading to a more pronounced kidney injury.^7^^,^^9^ The role of magnesium remains to be fully elucidated, as uptake of cisplatin into and out of the renal tubular cell is regulated by the copper transporter (Ctr1) and the organic cation transporter (Oct2). However, magnesium ions are recognized to compete with organic cations for the binding site in the channel pore of Oct2. So, if there is less magnesium, then more cisplatin can potentially be imported into the cell through Oct2, whereas the more the magnesium in the renal tubule, then the greater the competition for the transporter and so a reduction in cellular uptake of cisplatin.

A previous observational study of patients with head and neck cancer treated with low-dose weekly cisplatin reported hypomagnesemia after the sixth cycle of cisplatin treatment despite magnesium supplementation.^2^ In our study, we found that serum magnesium was significantly lower after the fifth week in the control group, whereas magnesium concentrations were maintained in the Mg group. Similarly, there was a sustained fall in eGFR after the fifth week in the control group, but not in the Mg group, suggesting that less renal tubular damage from cisplatin resulted in less tubular magnesium wasting and less kidney damage. The incidence of cis-AKI was not different between groups; however, the incidence of cis-AKD was higher and the time to cis-AKD was shorter in the control group. When considering changes in SCr from baseline, the increase was greater in the control group. However, one has to consider that other factors such as dietary intake and physical exercise can affect serum creatinine. One patient in the control group developed severe mucositis, which could have reduced dietary intake, while no patient was recorded as suffering from severe mucositis in the Mg group.

Our pilot study data underline the effects of cisplatin on kidney function which were mainly subacute, thus clinicians should carefully monitor not only for cis-AKI comparing pre- and post-dose SCr, but also for cis-AKD comparing post-dose SCr with the original pre-chemotherapy baseline SCr (Supplementary Material, https://doi.org/10.1016/j.esmoop.2021.100351).

More importantly, there have been concerns that treatments which reduce cisplatin nephrotoxicity might also reduce cisplatin uptake into tumor cells leading to reduce efficacy of cisplatin treatment. As magnesium in our study was given relatively rapidly as part of an intravenous volume load, this would lead to a forced diuresis with increased renal tubular magnesium losses, and so unlikely to have systemic effects outside the kidney. Reassuringly, animal models have shown that magnesium supplementation did not affect tumor cell killing activity,^7^^,^^9^ and clinical reports from studies reporting reduced cisplatin nephrotoxicity have not observed reduced effectiveness of chemotherapy in patients with lung and ovarian cancer.^11^^,^^12^ We also report that a magnesium-preloading regimen reduces the incidence of cis-AKD without reducing the chemotherapeutic effect of repeated low dose in patients treated for head and neck cancer. Furthermore, the long-term effects of cisplatin on kidney function such as CKD need to be elucidated in further studies.

### Conclusions

Our pilot study of magnesium-preloading regimen with 16 mEq significantly reduced cis-AKD without reducing the longer-term treatment efficacy of low-dose cisplatin chemotherapy in patients with head and neck cancer. Further large-scale randomized controlled trials are needed to confirm these encouraging results.
