# Supplementary figures and images for: Preloading magnesium attenuates cisplatin-associated nephrotoxicity: pilot randomized controlled trial (PRAGMATIC study)
Source: ESMO Open. 2021 Dec 23;7(1):100351. doi: 10.1016/j.esmoop.2021.100351 (PMC8717436; doi:10.1016/j.esmoop.2021.100351)

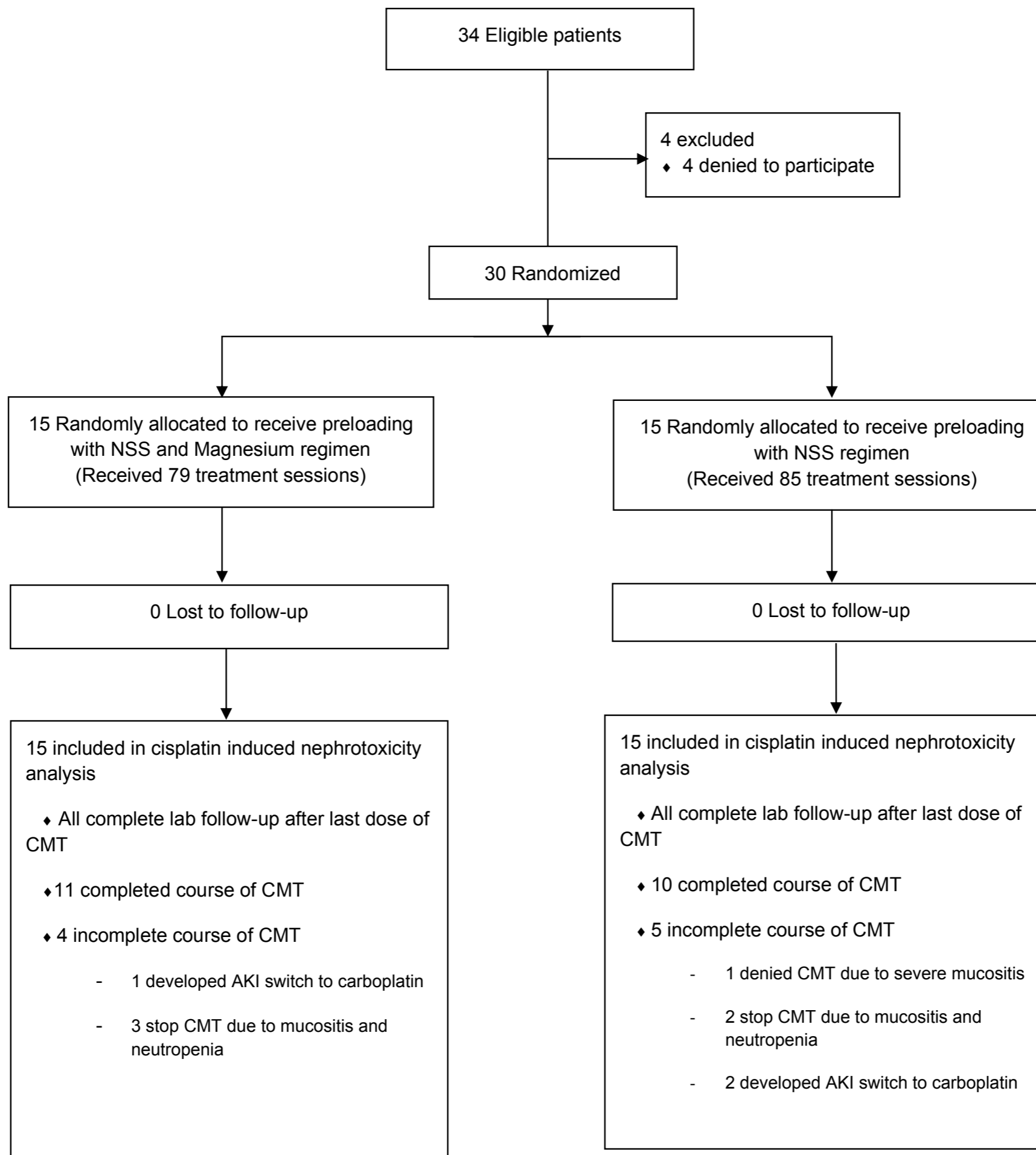

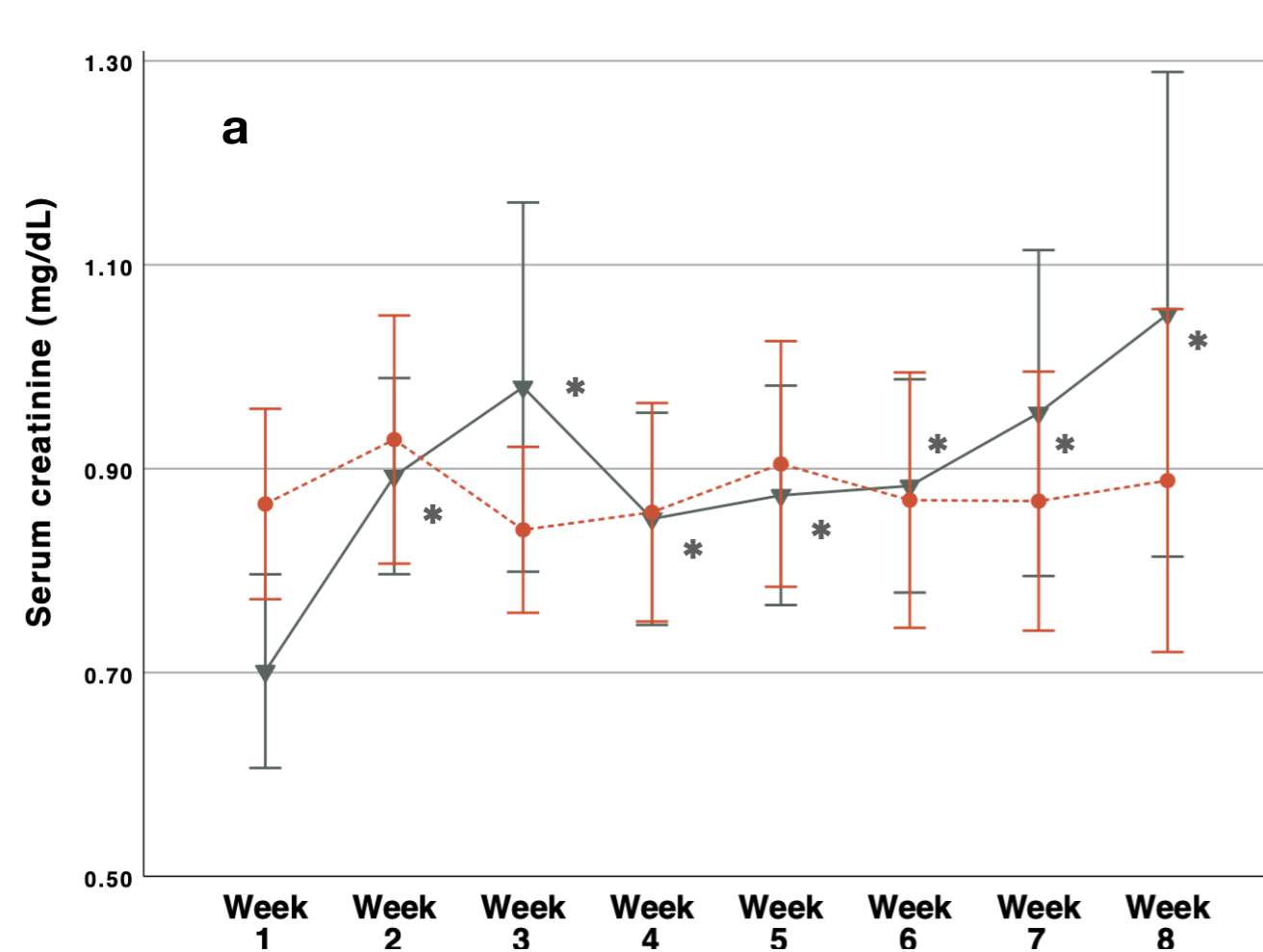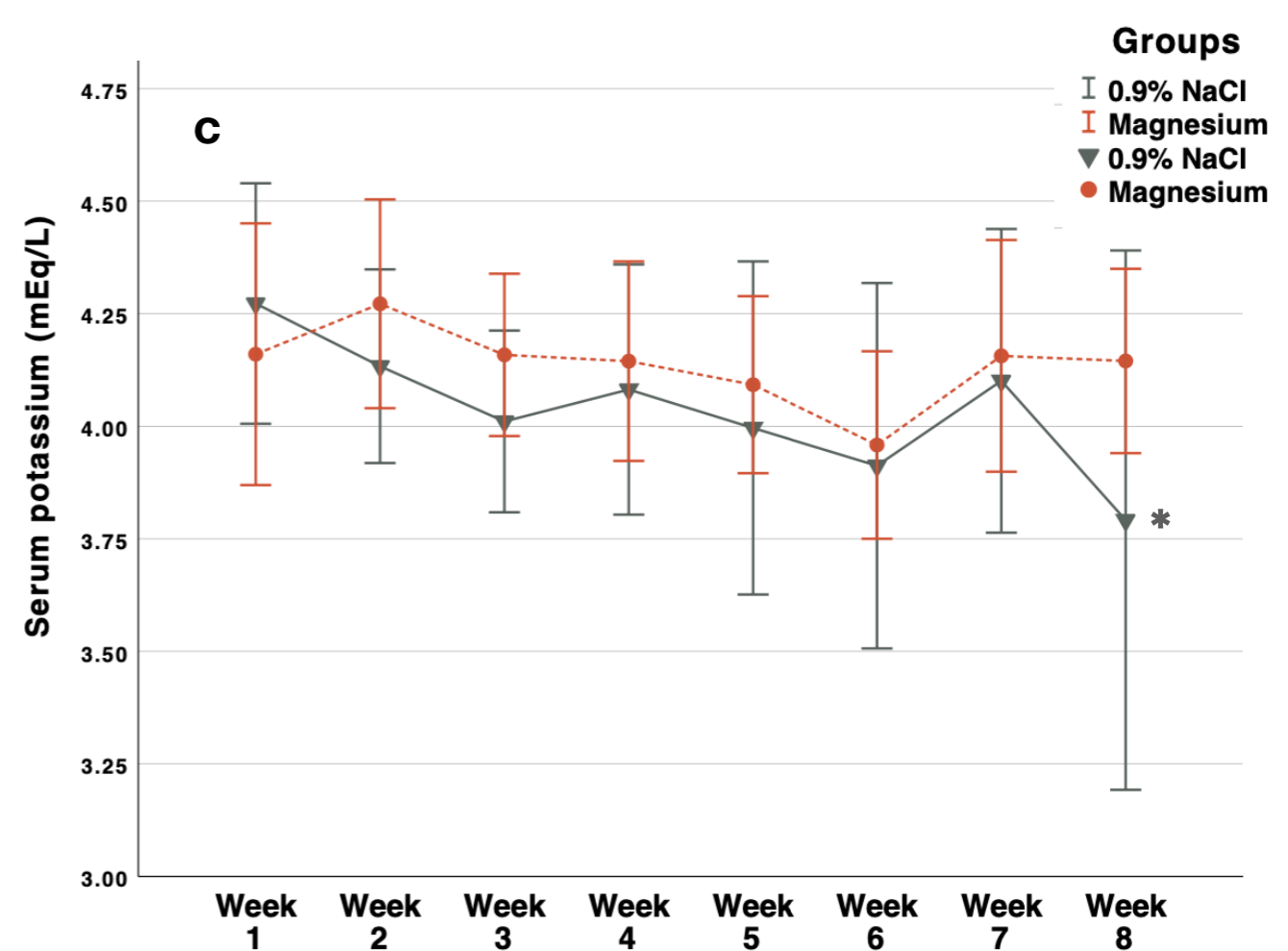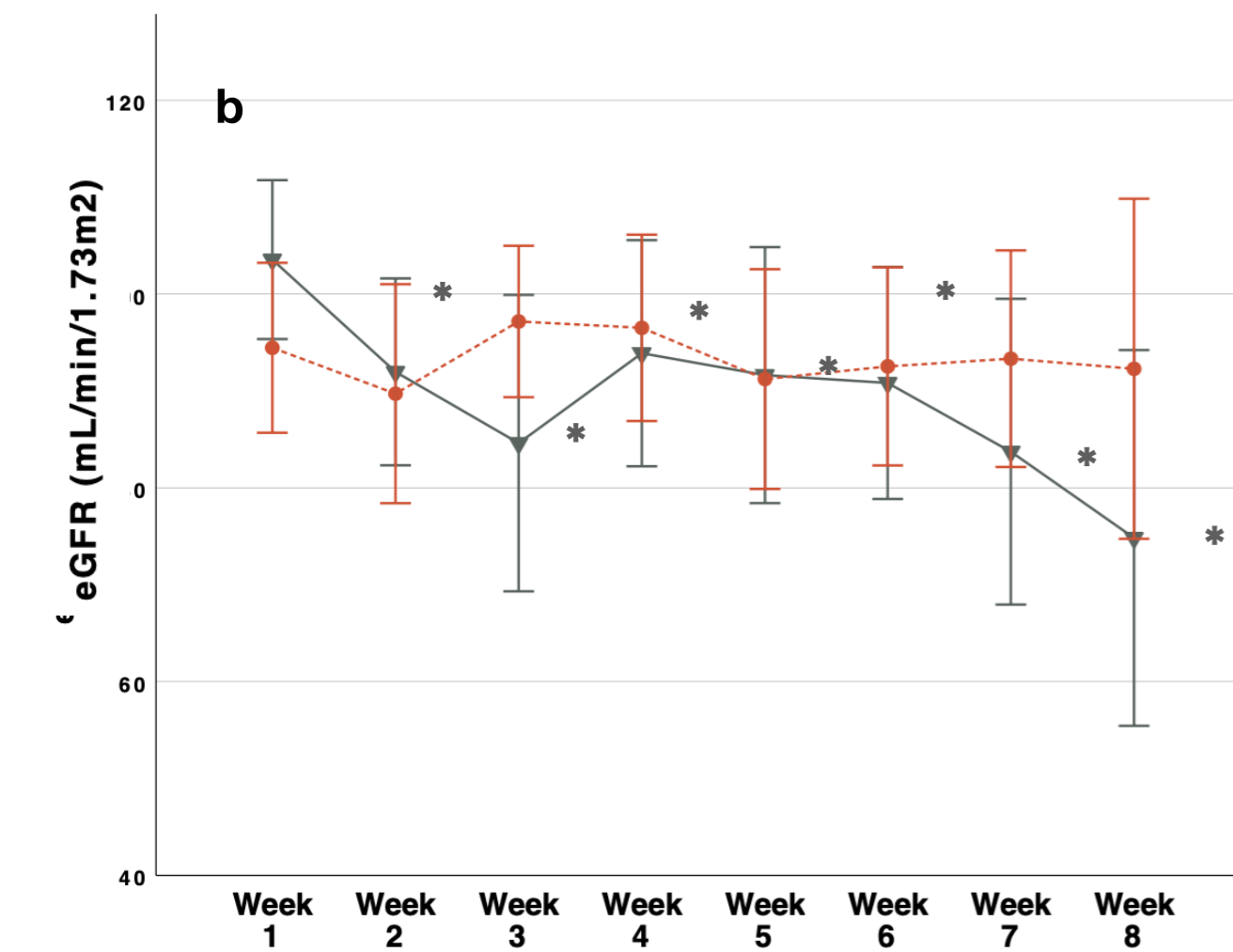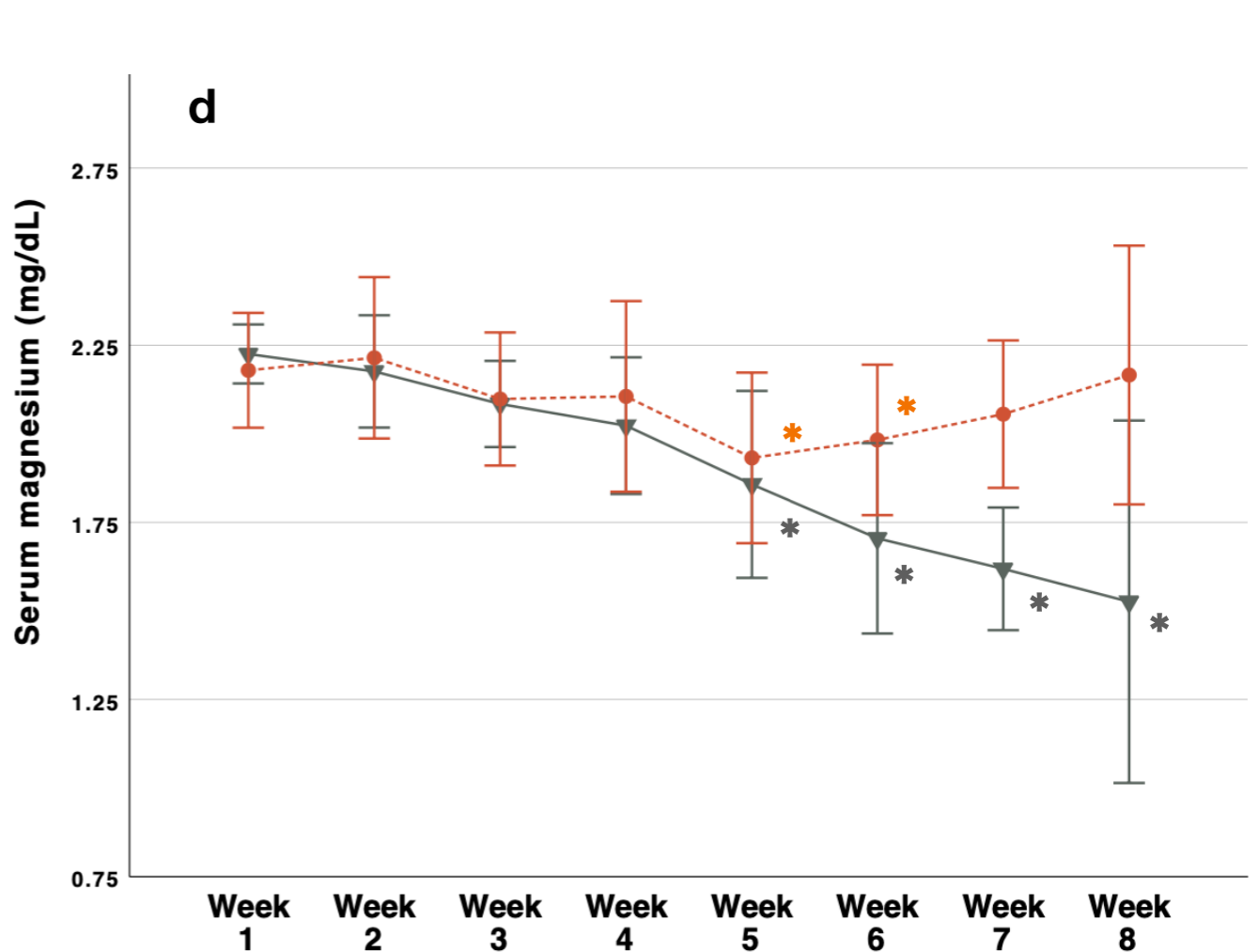

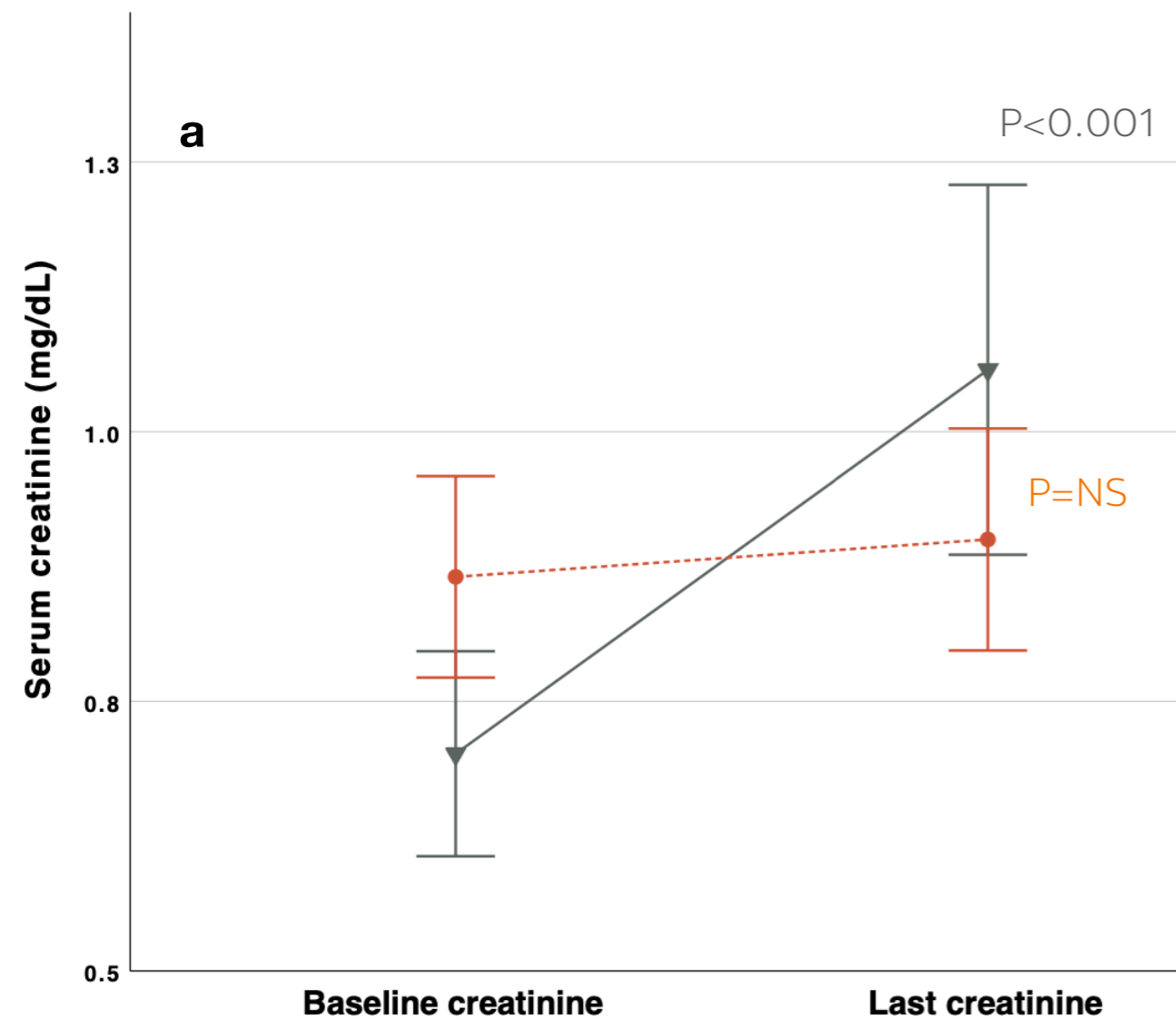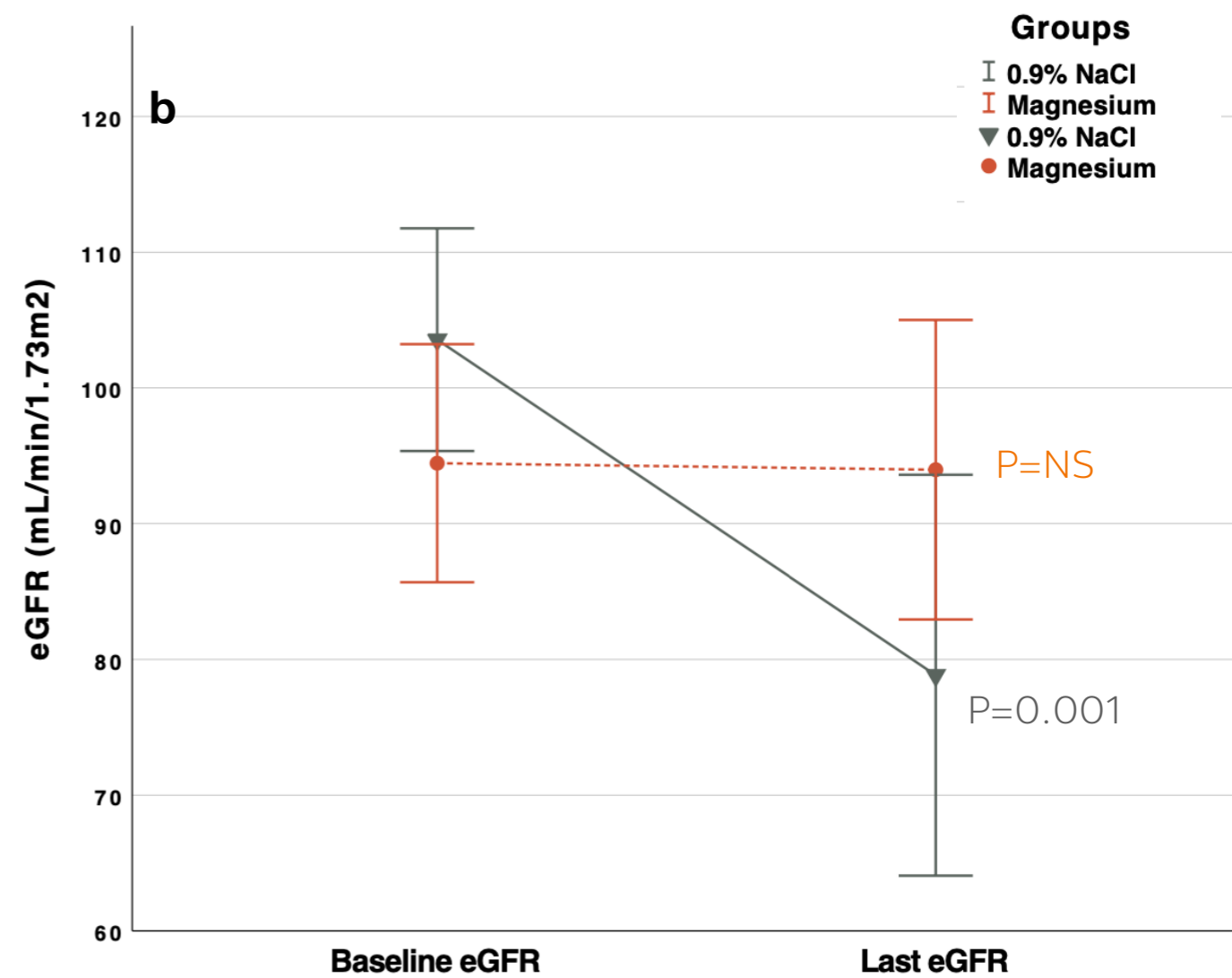

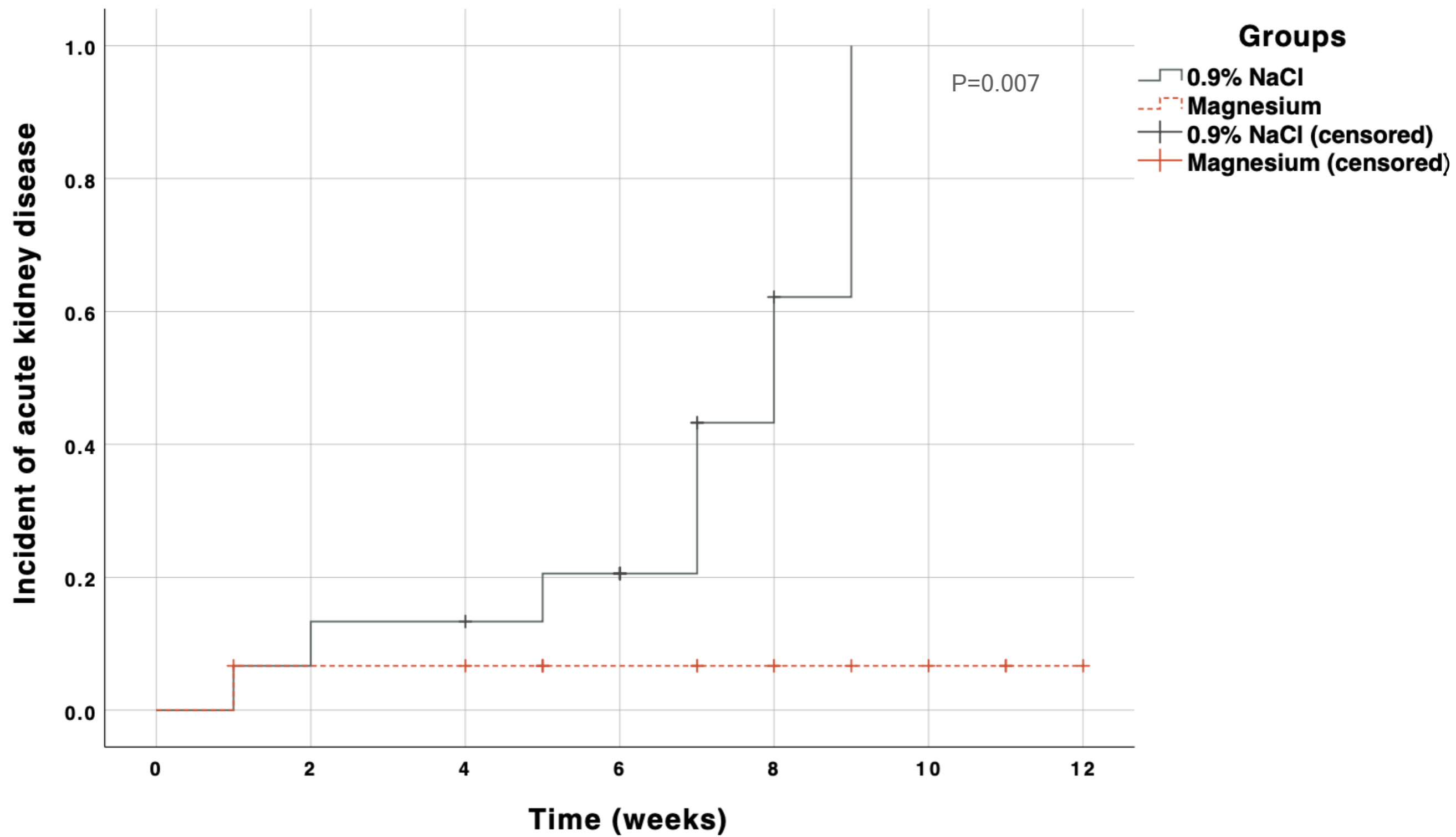

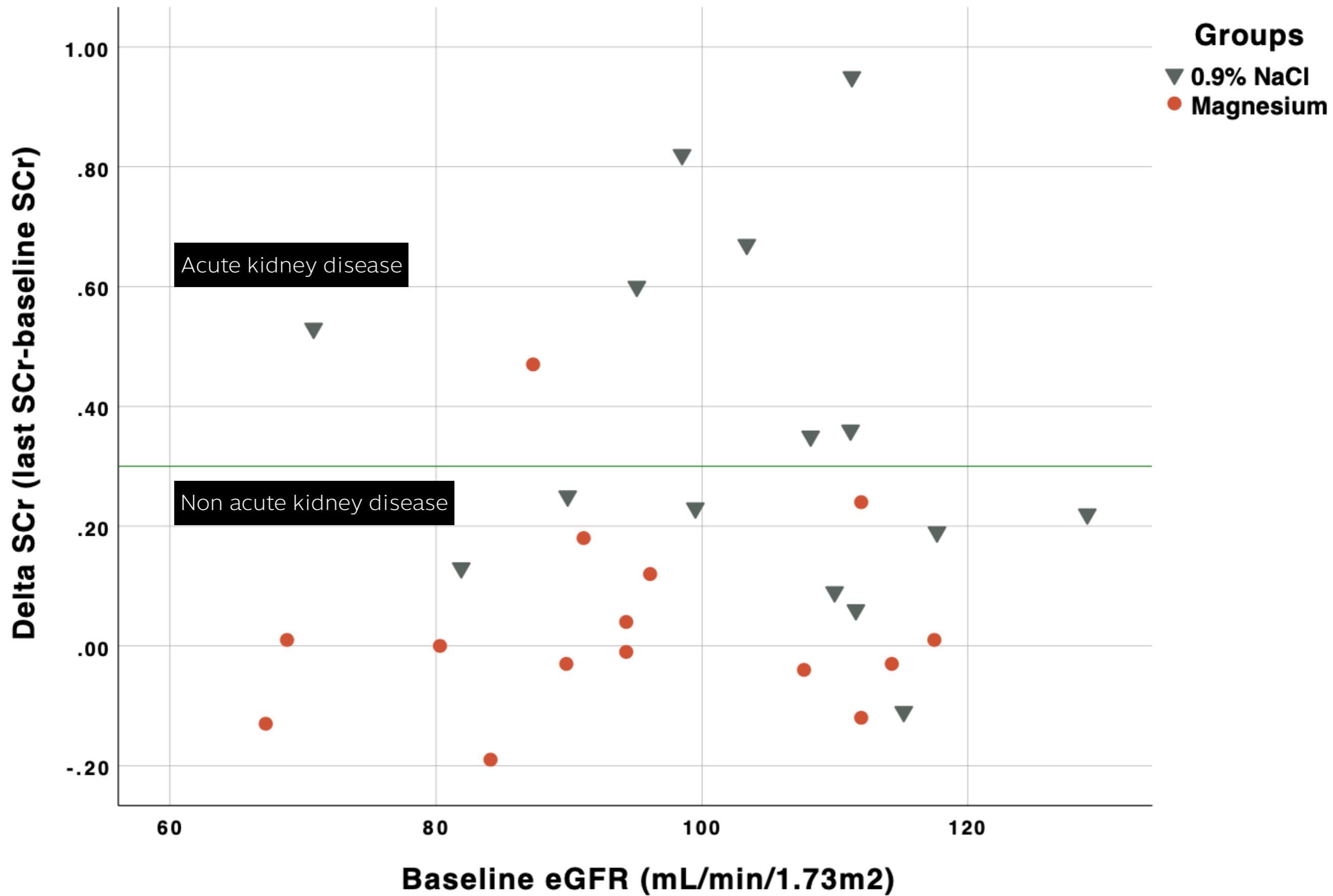

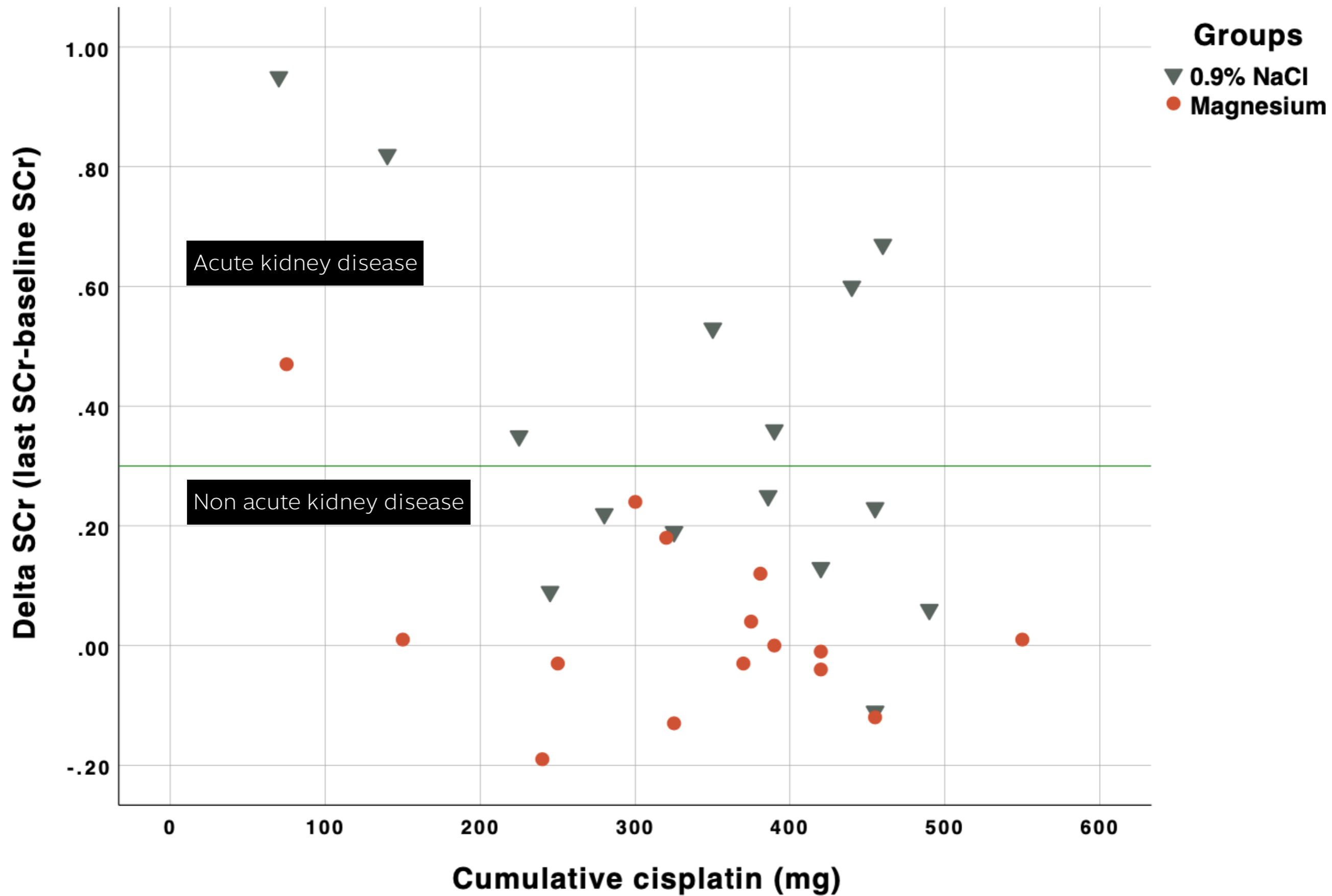

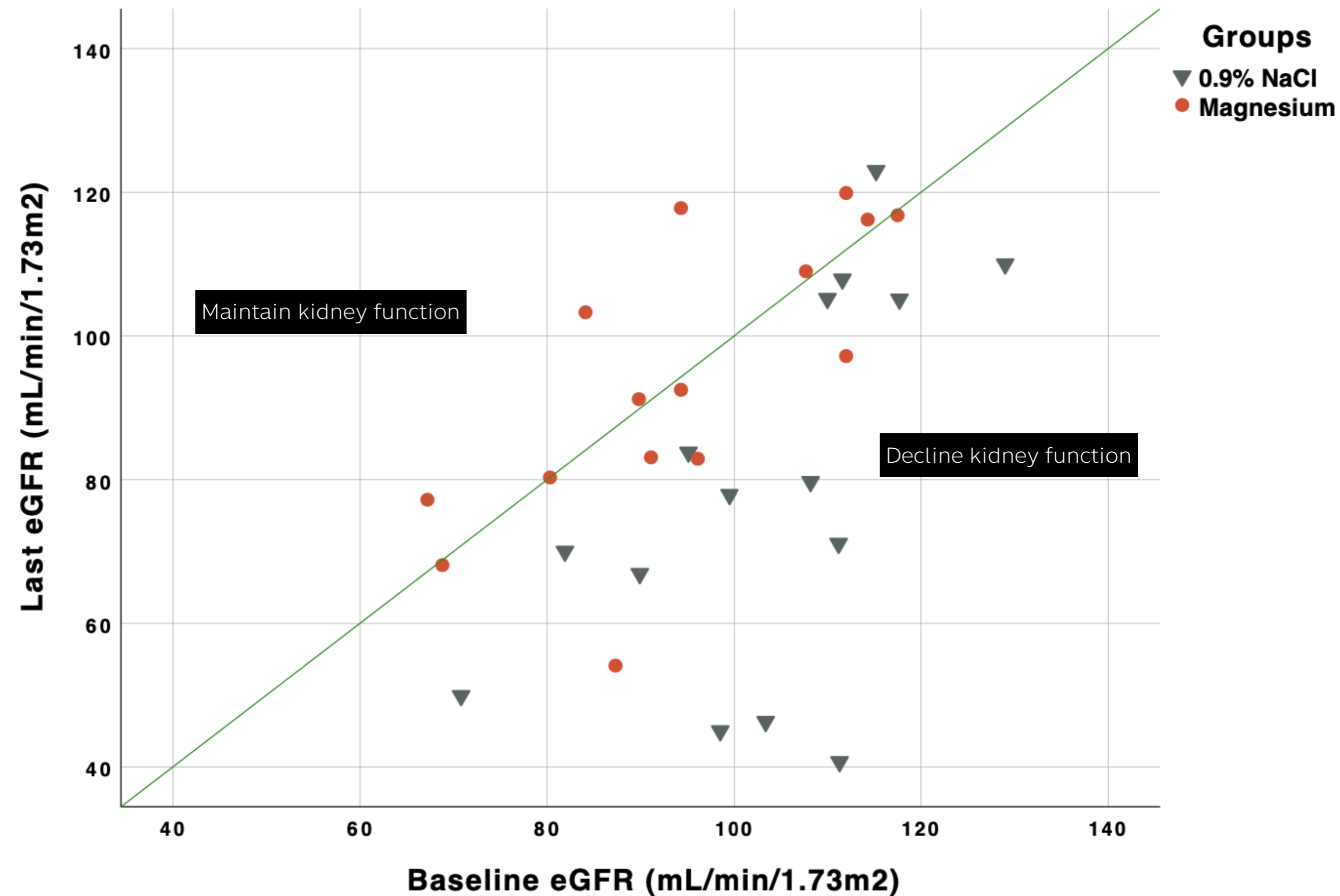

Supplement: Supplementary Figures [file mmc1.pdf]
