# Supplementary material for: Preloading magnesium attenuates cisplatin-associated nephrotoxicity: pilot randomized controlled trial (PRAGMATIC study)
Source: ESMO Open. 2021 Dec 23;7(1):100351. doi: 10.1016/j.esmoop.2021.100351 (PMC8717436; doi:10.1016/j.esmoop.2021.100351)
Supplement: Supplementary Figures [file mmc2.docx]

**Supplement figure 1.** Cumulative incident of acute kidney disease amongst treatment groups.

**Supplement figure 2.** Scatter plot showed correlation of acute kidney disease and baseline estimated glomerular filtration rate (eGFR) amongst treatment groups.

**Supplement figure 3.** Scatter plot showed correlation of acute kidney disease and cumulative dosage of cisplatin amongst treatment groups. SCr= Serum creatinine.

**Supplement figure 4.** Scatter plot showed correlation of baseline estimated glomerular filtration rate (eGFR) and last eGFR between the two treatment groups.
